# Targeting ErbB3-mediated stromal–epithelial interactions in pancreatic ductal adenocarcinoma

**DOI:** 10.1038/bjc.2011.263

**Published:** 2011-07-26

**Authors:** J S Liles, J P Arnoletti, A V Kossenkov, A Mikhaylina, A R Frost, P Kulesza, M J Heslin, A Frolov

**Affiliations:** 1 Department of Surgery, University of Alabama at Birmingham, Birmingham, AL 35294, USA; 2 Systems Biology Division, The Wistar Institute, Philadelphia, PA, USA; 3 Department of Pathology, University of Alabama at Birmingham, Birmingham, AL 35294, USA; 4 Department of Pathology, Northwestern University, Chicago, IL, USA

**Keywords:** ErbB3, EGFR, pancreatic cancer, ErbB3 antibody, tumourigenesis

## Abstract

**Background:**

We sought to investigate the role of ErbB3-mediated signalling on the interaction between pancreatic cancer-associated fibroblasts (CAF) and carcinoma cells in an effort to disrupt tumourigenic pancreatic ductal adenocarcinoma (PDAC) stromal–epithelial cross-communication.

**Methods:**

Primary CAF cultures were established from human PDAC surgical specimens. AsPC-1 pancreatic cancer cell murine subcutaneous xenografts were developed in the presence and absence of CAF and were subsequently treated with epidermal growth factor receptor (EGFR) inhibitors (erlotinib) and ErbB3 inhibitors (MM-121, monoclonal ErbB3 antibody).

**Results:**

Cancer-associated fibroblasts were found to secrete neuregulin-1 (NRG-1), which promoted proliferation via phosphorylation of ErbB3 and AKT in AsPC-1 PDAC cells. This signalling cascade was effectively inhibited both *in vitro* and *in vivo* by specific ErbB3 blockade with MM-121, with greater degree of tumourigenesis inhibition when combined with erlotinib. The CAF–AsPC-1 pancreatic cancer xenografts reached significantly greater tumour volume than those xenografts lacking CAF and were resistant to the anti-tumour effects of EGFR inhibition with erlotinib.

**Conclusion:**

Cancer-associated fibroblasts-derived NRG-1 promote PDAC tumourigenesis via ErbB3-AKT signalling and overcomes single-agent EGFR inhibition. Disruption of this stromally mediated tumourigenic mechanism is best obtained through combined EGFR-ErbB3 inhibition with both erlotinib and MM-121. We have identified the NRG-1/ErbB3 axis as an attractive molecular target for the interruption of tumourigenic stromal–epithelial interactions within the PDAC microenvironment.

Pancreatic ductal adenocarcinoma (PDAC) is histologically characterised by an expansive, dense fibrotic reaction known as desmoplasia. This tumour-associated stroma surrounds the ductal adenocarcinoma cells and is composed of extracellular matrix proteins, fibroblasts and stellate cells, adipocytes, endothelial and immune cells. Although a desmoplastic reaction is found in other epithelial tumours, the stromal exuberance associated with PDAC is unique. After being overlooked for years, recent evidence suggests that this desmoplasia may actively support carcinoma cells by providing a reactive microenvironment that dynamically secretes growth factors and cytokines, inhibits tumour cell apoptosis and promotes resistance to therapeutic agents (Mahadevan and Von Hoff, 2007; Bachem *et al*, 2008; Hwang *et al*, 2008).

The epidermal growth factor receptor (EGFR) signal transduction system and its associated cell-surface receptors is one of the most studied tyrosine kinase signalling networks in cancer. Epidermal growth factor receptor overexpression has been demonstrated in a wide range of epithelial malignancies, and in many of these tumours, has been shown to correlate with poor prognosis (Modjtahedi and Essapen, 2009). It has been thoroughly demonstrated *in vivo* that activation of this receptor family in malignant cells results in reduced apoptosis and increased proliferation, motility, invasion and metastasis. Several anti-EGFR agents, including monoclonal antibodies and small molecule tyrosine kinase inhibitors have been approved by the US Food and Drug Administration for the treatment of patients with advanced epithelial tumours, including non-small-cell lung cancer (NSCLC), colorectal, head and neck, pancreatic and breast cancer (Modjtahedi and Essapen, 2009).

In the case of PDAC, results from *in vitro* and animal experiments showed much promise for EGFR-targeting agents, but clinical trials have demonstrated modest improvement in overall patient survival (Moore *et al*, 2007). Subset analysis has shown, however, that patients who develop skin rash seem to obtain the greatest benefit from this type of treatment supporting a role for therapeutic strategies based on anti-EGFR agents in PDAC (Wacker *et al*, 2007). Carefully designed clinical studies have yet to reliably identify those patients who are more likely to have a favourable response as the underlying mechanisms of resistance remain poorly understood.

We have previously demonstrated that ErbB3, a member of the EGFR family of receptor tyrosine kinases, has a pivotal role in pancreatic tumourigenesis through heterodimerisation with EGFR and induction of phosphoinositide 3-kinase (PI3K)/AKT signalling, which promotes *in vitro and in vivo* cancer cell proliferation (Liles *et al*, 2010). The role of ErbB3 as a highly specialised activator of other ErbB receptors has only recently been acknowledged. The ErbB3 cell-surface receptor, historically overlooked due to its perceived lack of catalytic activity, has been thought to be inert, functioning only as a scaffolding partner for other more active receptors. It is now known that ErbB3 contains a kinase domain which, when exposed, binds to other ErbB receptors resulting in their activation (Zhang *et al*, 2006; Jura *et al*, 2009). Additional findings from our laboratory also point to the lack of activating mutations in *EGFR* and *ErbB3* genes, giving further strength to ligand-driven tumour cell proliferation as a paramount tumourigenic mechanism within the PDAC microenvironment (Tzeng *et al*, 2007a). Current progress in understanding the important role of ErbB3 has sparked the development of clinical anti-ErbB3 agents. In this study we used MM-121, a fully humanised anti-ErbB3 antibody currently under clinical development, which has been shown to be active in other tumour types.

The purpose of our study was to analyse the role of cancer-associated stroma in PDAC progression while delineating the effects of targeted ErbB3 inhibition in combination with anti-EGFR therapy. We intended to better characterise stromally secreted ligands that induce ErbB signalling in the neighbouring pancreatic carcinoma cells. We hypothesised that cancer-associated fibroblasts (CAF) secrete NRG-1 ligand, which in turn activates cancer cell ErbB3/AKT-mediated signalling, promoting tumourigenesis and rendering EGFR inhibition ineffective. Finally, we wanted to determine if targeted ErbB3 inhibition could effectively prevent tumour cell proliferation and downstream signalling in PDAC.

## Materials and methods

### Reagents

Erlotinib (generously provided by OSI Pharmaceutical Inc., Melville, NY, USA) was dissolved in DMSO to prepare a 20-mM stock solution. MM-121 (generously provided by Merrimack Pharmaceutical, Cambridge, MA, USA) was dissolved directly in the medium to achieve a final concentration of 250 *μ*g ml^−1^. Epidermal growth factor (1 mg ml^−1^) and heregulin-*β*1, EGF-like domain (neuregulin-*β*1; 1 mg ml^−1^) were purchased from Sigma (St Louis, MO, USA). ErbB3 blocking/receptor neutralising peptide (clone H3.105.5, Millipore, Temecula, CA, USA) was added directly to the medium to achieve a final concentration of 10 *μ*g ml^−1^. Anti-NRG-1 antibody (sc-1793, Santa Cruz Biotechnology, Santa Cruz, CA, USA) was added directly to the medium to achieve a final concentration of 50 *μ*g ml^−1^. Drug concentrations used for *in vitro* and *in vivo* studies were based on published data, communications with the manufacturers and our previous work (Jimeno *et al*, 2006; Frolov *et al*, 2007; Schoeberl *et al*, 2010).

### Cell culture

All cell lines were propagated in a humidified atmosphere containing 5% CO_2_ at 37°C. Pancreatic cancer cell lines were purchased from the American Type Culture Collection (Rockville, MD, USA) and propagated according to the provider's recommendations. S2VP10 cells were generously provided by Dr Michael Hollingsworth (University of Nebraska).

### Proliferation assay

Cells were seeded in 96-well plates (1.5 × 10^3^ cells per well), allowed to propagate overnight before treatment with erlotinib (5 *μ*M) or MM-121 (250 *μ*g ml^−1^, suggested by the Merrimack) and stimulated with either EGF (0.1 *μ*g ml^−1^) or NRG-1*β* (0.22 *μ*g ml^−1^). CellTiter 96 AQueous One Solution Cell Proliferation Assay (Promega, Madison, WI, USA) was used to assess absolute proliferation in cell lines or relative proliferation defined as a ratio between the proliferation of the control untreated cells and drug-treated cells.

### Pancreatic cancer patient specimens and laser-capture microdissection

After Institutional Review Board (IRB)-approved informed consent was obtained, tumour specimens were collected from PDAC patients who underwent surgical treatment under the guidelines of the Surgical Oncology Tumor Bank. Specimens were snap frozen at the time of operation and stored at −80°C. Laser-capture microdissection (LCM) was performed as previously described (Tzeng *et al*, 2007a). An expert pancreatic pathologist (PK) evaluated each specimen and identified regions of adenocarcinoma cells and stromal cells before dissection. Approximately 3000 tumour cells were captured from each specimen for analysis.

### RNA extraction and reverse transcriptase PCR

RNA from microdissected patient specimens was isolated and converted to cDNA as previously described (Liles *et al*, 2010). Quantitative real-time PCR was performed using TaqMan Gene Expression *EGFR*, *ErbB3*, *EGF*, *TGF-α*, *NRG-1α*, *NRG-1β* and *RPLPO* (housekeeping gene) Assays-on-Demand and TaqMan Universal PCR Master Mix in an ABI Prism 7700 Detection System (Applied Biosystems, Carlsbad, CA, USA). Reverse transcriptase PCR (RT–PCR) data are the average of triplicate experiments and represent expression value relative to *RPLPO* expression in the same specimen.

### Western blotting

Protein lysates were prepared from cell lines or pulverised frozen tumours and standard SDS–PAGE, western blotting and chemiluminescence were performed as previously described (Frolov *et al*, 2007). The following antibodies were obtained from Millipore: anti-pEGFR tyr845 and anti-pEGFR tyr1173; Sigma: anti-*β*-actin and anti-EGFR; Cell Signaling (Beverly, MA, USA): anti-pEGFR tyr992, anti-pEGFR tyr1068, anti-pErbB3 tyr1289, anti-ErbB3, anti-pAKT ser473, anti-AKT, anti-pp44/42 MAPK thr202/tyr204 and anti-MAPK. All antibodies were used as specified by the manufacturer and diluted in 5% milk. Western blot quantification was performed using ImageJ software package (NIH, Bethesda, MD, USA) according to the developer's instructions.

### Primary fibroblast cultures

Human PDAC specimens were obtained in the operating room as part of patient treatment protocols at our institution and tumour sections were collected under the guidelines of an IRB-approved protocol. Specimens were minced and allowed to incubate in serum-supplemented media (10% FBS in RPMI media) for 48–72 h, after which time, media was replaced and CAF were collected. Fibroblasts were further propagated in DMEM/F12 media supplemented with 20% FBS. Cancer-associated fibroblasts used in the *in vivo* model were immortalised by hTERT expression. Full-length hTERT in a pGIPZ expression vector was obtained from Thermo Fisher Scientific (Pittsburgh, PA, USA).

### Primary fibroblasts conditioned media

Primary fibroblast cultures were grown to 70% confluence. Culture media was replaced with serum-free media and was incubated with cells for an additional 48 h. Cell-conditioned media was then collected, filtered and concentrated using either 3 or 30 kDa cutoff bioseparation devices (Millipore). Cell-conditioned media was analysed immediately and no freeze-thaw cycles were allowed.

### Immunohistochemistry

Cells were grown on cover slips as described above. Cover slips were then fixed and stained for cytokeratin-5, cytokeratin-8, pErbB3, epithelial membrane antigen (EMA), *α*-smooth muscle actin (*α*-SMA), desmin and vimentin at the UAB Clinical Pathology core facility using standard immunohisotchemistry techniques. Stained slides with appropriate negative and positive controls were reviewed by an expert cytopathologist (PK). For xenografts, serial 5 *μ*m sections were cut 1 day before immunostaining from the representative formalin-fixed and paraffin-embedded blocks and mounted on Superfrost/Plus slides (Fisher Scientific). Sections were then incubated overnight with primary monoclonal antibodies for pErbB3 and *α*-SMA (Santa Cruz Biotechnology). Secondary detection was performed using a multi-species detection system (Signet Lab Inc., Dedham, MA, USA). Sections were incubated in biotinylated anti-mouse antibodies for 20 min, followed by peroxidase-labelled streptavidin for 20 min (Signet Lab Inc.). Antigen–antibody complexes were visualised by incubation with 3,3′-diaminobenzidine substrate (BioGenex, San Ramon, CA, USA) and counterstained with diluted Harris haematoxylin. The stained slides were systematically evaluated by a pathologist. The scoring for *α*-SMA was performed by estimating the fraction of positive cells by cell number (0–100%), and comparing spindle *vs* epithelial cell morphology. The pErbB3 scoring was performed by estimating the fraction of positive epithelial cells only, and multiplying by an arbitrary, discrete intensity scale where 0 is negative, and 3 is strongest positive. All negative control slides (omitted primary antibodies) were negative for staining.

### Murine xenografts

Six-week-old female in-bred Fox Chase SCID mice were obtained from Charles River Laboratories (Hartford, CT, USA). Animals were handled according to a protocol approved by the Institutional Animal Care and Use Committee at our university. Mice were allowed to acclimate to animal housing, and xenografts were developed by subcutaneously injecting 5 × 10^6^ AsPC-1 cells with or without primary fibroblasts (5 × 10^6^ cells for 1:1 CAF–AsPC-1 cell ratio and 1 × 10^7^ cells for 2 : 1 CAF–AsPC-1 cell ratio) to the murine flank bilaterally. Trice weekly, tumour volume was determined using digital caliper measurements and the formula: 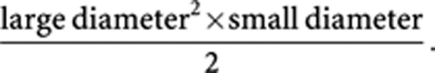


After 14 days, all mice had measurable tumours and were sorted into treatment and control groups with equal number of animals (*n*=5). Treatment group mice received 50 mg kg^−1^ erlotinib dissolved in vehicle (0.1 M NaCl, 0.05% pluronic acid in PBS) per treatment while control mice received vehicle only. All mice received 10 intraperitoneal injections over a 14-day period (cycle: five treatment days followed by two non-treatment days). After 14 days, mice were killed.

### Statistical analysis

Correlations between mRNA levels were compared by Mann–Whitney rank test or two-tailed *t*-test employing SPSS software (Chicago, IL, USA). All cell assay conditions were performed in six wells and repeated in triplicate independent runs and data are presented as mean±s.e.m. In these assays, Student's *t*-test was used for comparison and the Mann–Whitney *U*-test was used to compare independent runs. Rate of *in vivo* tumour growth for a replicate was calculated as a slope of regression line fitted to data representing tumour volume *vs* seven time points of treatment. Mean rate for a group was calculated as an average of all rates for the group. Rates between any two groups were compared using paired two-tailed *t*-test. Statistical significance level was set at *P*-value <0.05.

## Results

### *NRG-1* is preferentially expressed in the PDAC stromal fibroblast compartment

**Figure 1:**
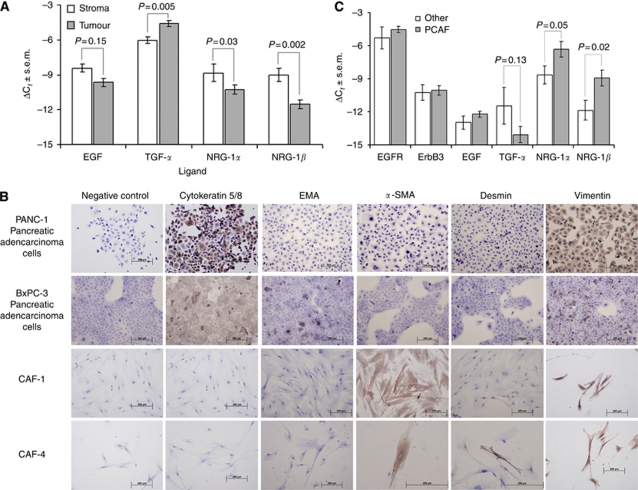
(**A**) Ligand expression differences between stroma and tumour in microdissected pancreatic cancer samples. ΔC_*t*_ values were calculated as a difference between the target gene C_*t*_ and *RPLPO* (housekeeping gene) C_*t*_ values (C_*t*_=threshold cycle). Bars represent mean values±s.e.m. *P*=*P*-value for Mann–Whitney rank test. (**B**) IHC analysis of two primary CAF cultures CAF-1 and CAF-4 together with Panc-1 and BxPC-3 cell lines. (**C**) Gene expression differences between CAF and normal tissue fibroblasts. ΔC_*t*_ values are calculated against RPLPO C_*t*_ values. Bars represent mean values±s.e.m. *P*=*P*-value for two-tailed *t*-test.

We used LCM of surgically resected pancreatic cancer specimens (*n*=42) to selectively isolate PDAC cells and their surrounding CAF and to characterise their respective contributions to ligand-induced ErbB signalling within the PDAC microenvironment. We analysed mRNA transcript expression levels of EGFR ligands (*EGF* and *TGF-α*) and two isoforms of the ErbB3 ligand, NRG-1 (*NRG-1α* and *NRG-1β*) in each one of those two tumour compartments (Figure 1A). *TGF-α* expression was significantly higher in the ductal adenocarcinoma cells than in CAF (*P*=0.005), and EGF was equally expressed by both stroma and epithelial cancer cells. Conversely, the two isoforms of NRG-1 were expressed at significantly higher levels in the stromal CAF (*NRG-1α*, *P*=0.03; *NRG-1β*, *P*=0.002). Interestingly, *NRG-1β* expression was 2–3 times greater in the stromal fibroblast compartment when compared with carcinoma cells, suggesting that this ligand is preferentially produced in the stroma and exerts paracrine activity on carcinoma cells.

### Establishment of primary fibroblast cultures and analysis of ErbB ligand expression

To further characterise stromal sources of ErbB ligands, we established primary CAF cultures from fresh human pancreatic cancer specimens (*n*=23). Although the morphological appearance of the isolated CAF indicated they were fibroblasts, immunohistochemical analysis was performed for lineage verification. Cultured primary CAF (two representative cell lines, CAF-1 and CAF-4, were employed in this experiment) expressed desmin, *α*-SMA and vimentin, which confirmed their mesenchymal lineage (Figure 1B) and clearly distinguished them from epithelial pancreatic cancer cell lines (PANC-1 and BxPC-3), which display cytokeratin and EMA. Expression of vimentin by some pancreatic carcinoma cells results from underlying epithelial-to-mesenchymal transition, a phenomenon previously described in this type of tumour cell (Buck *et al*, 2007).

In addition, fibroblasts from different tissue sources (*n*=9) including normal pancreas, normal dermis, chronic pancreatitis, gastrointestinal stromal tumour, pheochromocytoma and colon carcinoma were isolated. RNA was extracted and pooled with the intent of comparing levels of ErbB ligand and ErbB receptor expression to those of pancreatic CAF. Analysis of mRNA transcript levels in these cells demonstrated that fibroblasts are distinctly different in their expression of ErbB ligands based on their tissue of origin. We found significantly higher levels of *NRG-1α* and *NRG-1β* mRNA expression in pancreatic CAF (*P*=0.05 and *P*=0.02, respectively) relative to all other isolated fibroblasts (Figure 1C). Interestingly, ErbB receptor expression (*EGFR* and *ErbB3* mRNA transcript levels) was not significantly different among the different types of analysed fibroblasts (data not shown).

### CAF-conditioned media contains NRG-1 and induces AKT signalling and cellular proliferation *in vitro*

**Figure 2:**
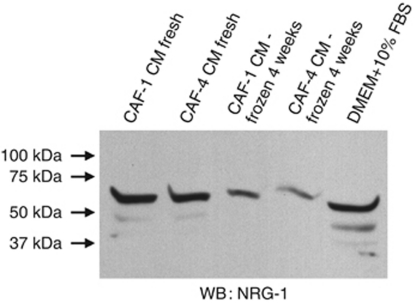
Western blot analysis of NRG-1 secretion into CAF-conditioned media filtered with 30 kDa cutoff filter (both fresh and after a single freeze-thaw cycle).

**Figure 3:**
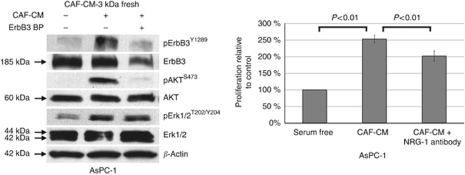
Western blot and proliferation analysis of CAF-conditioned media effects on AsPC-1 cells. Addition of ErbB3 blocking peptide or anti-NRG-1 neutralising antibody partially abrogates the stimulating effect of CAF-CM.

To evaluate the postulated paracrine signalling mechanism of CAF on tumour cell growth, we collected serum-free CAF cell-conditioned media (CAF-CM). The CAF-CM was selectively filtered with a 3-kDa and a 30-kDa cutoff filter in order to concentrate the media and remove debris. Western blot analysis of CAF-CM confirmed expression of NRG-1 in both the 3- and 30-kDa filtered CAF-CM. We also demonstrated that NRG-1 deteriorates with freeze-thaw cycles, therefore, requiring fresh CAF-CM for all experiments (Figure 2). Stimulation of AsPC-1 cells with CAF-CM resulted in phosphorylation of ErbB3 and AKT, and this intracellular signalling effect was prevented by blocking the receptor with an ErbB3-binding/receptor neutralising peptide (Figure 3). CAF-CM promoted pancreatic cancer cell proliferation *in vitro* and this effect was moderately diminished by a presence of the inhibitory NRG-1 antibody (*P*<0.01) (Figure 3). The modest effect of the inhibitory NRG-1 antibody could be explained by a modest binding ability of the antibody as well as ongoing proliferation promotion exerted by additional cytokines in the CAF-CM. Nevertheless, our findings confirm without a doubt the presence of CAF-secreted NRG-1 and the stimulatory effects of this ligand on ErbB3 signalling and proliferation of pancreatic cancer cells, further supporting the hypothesis of active stromal–epithelial interaction in PDAC.

### NRG-1*β* rescues pancreatic cancer cells from erlotinib inhibition *in vitro*

**Figure 4:**
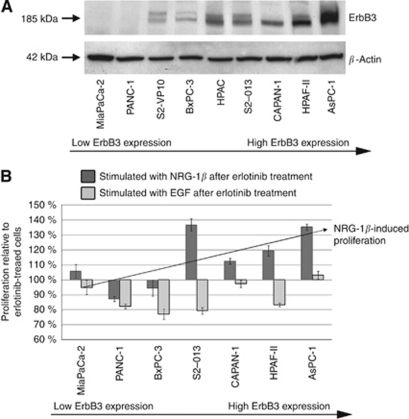
NRG-1*β* abrogates the inhibitory effect of erlotinib *in vitro*. (**A**) ErbB3 expression analysis of nine representative pancreatic cell lines. (**B**) Relative proliferation of nine pancreatic cell lines treated with erlotinib with subsequent stimulation with either EGF or NRG-1*β*. Proliferation of the erlotinib-treated control cells is set at 1.0 on the *y* axis.

To further investigate the potential influence of ErbB ligands on response to EGFR-targeted therapy, we treated seven PDAC cell lines with erlotinib followed by ligand stimulation with EGF and/or NRG-1*β*. We have previously demonstrated that four of these cell lines all express high levels of EGFR protein while HPAC, MiaPaCa-2 and CAPAN-1 express intermediate amounts of EGFR. We have also described the individual sensitivity of each cell line to erlotinib, with Panc-1 and MiaPaCa-2 cells, displaying resistance to the anti-proliferative effects of the drug (Frolov *et al*, 2007). Western blot analysis revealed variable baseline ErbB3 protein expression levels in the analysed cell lines (Figure 4A). Pancreatic ductal adenocarcinoma cells that display relatively high expression of ErbB3 (S2-013, CAPAN-1, HPAF-II and AsPC-1), overcome erlotinib-induced growth inhibition when exposed to NRG-1*β* (Figure 4B). Of interest, NRG-1*β* failed to stimulate proliferation in the three cell lines with undetectable or very low ErbB3 protein expression (MiaPaCa-2, PANC-1 and BxPC-3). As expected, baseline levels of pancreatic cancer cell EGFR protein did not correlate with the extent of erlotinib-induced growth inhibition.

**Figure 5:**
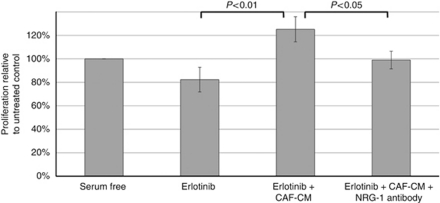
Addition of neutralising NRG-1 Ab limits proliferative escape from erlotinib-induced inhibition in the presence of CAF-conditioned media.

In an attempt to replicate the effects of exogenously added NRG-1, AsPC-1 PDAC cells were treated with erlotinib in the presence of CAF-CM. The erlotinib-induced inhibitory effect was completely abrogated when AsPC-1 cells were exposed to CAF-CM. As described above, CAF-CM contains NRG-1 and its effects closely resemble those of exogenous NRG-1. This fact was further confirmed when the protective effect of CAF-CM was partially blocked by pre-incubating the CAF-CM with an inhibitory NRG-1 antibody (Figure 5). In other words, CAF-secreted NRG-1 reverted the inhibitory effects of erlotinib on PDAC cell proliferation, suggesting a NRG-1-mediated escape mechanism. We attributed the modest growth inhibition seen with erlotinib in this case to the serum-free conditions of the experiment.

### CAF promote pancreatic cancer tumourigenesis

**Figure 6:**
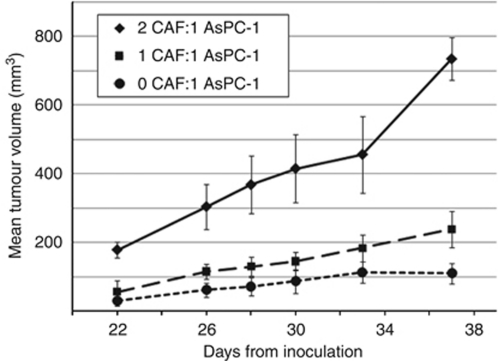
AsPC-1 tumour growth without CAF (•) and with CAF at 1 : 1 ratio (▪) and 1 : 2 ratio (▴).

We generated CAF-containing AsPC-1 xenografts to determine the effects of CAF on pancreatic cancer tumourigenesis. Analysis of human PDAC specimens determined that the ratio of stromal fibroblasts to carcinoma cells ranges from 2 : 1 to 1 : 1 (data not shown, Dr Andra Frost, personal communication). Therefore, similar ratios of CAF to AsPC-1 cells were employed for our xenografts experiments. After a 4-week interval after injection, higher CAF:AsPC-1 cell ratio directly correlated with greater tumour volume (*P*<0.001) (Figure 6). Xenografts with a 2 : 1 ratio of CAF:AsPC-1 had a significantly greater average tumour volume than xenografts with a 1 : 1 ratio and a 0 : 1 ratio (*P*<0.01). The possibility of CAF-induced tumourigenesis in the absence of carcinoma cells was ruled out by the absence of tumour development in a control group of animals inoculated with CAF only.

### CAF promote resistance to erlotinib therapy *in vivo*

**Figure 7:**
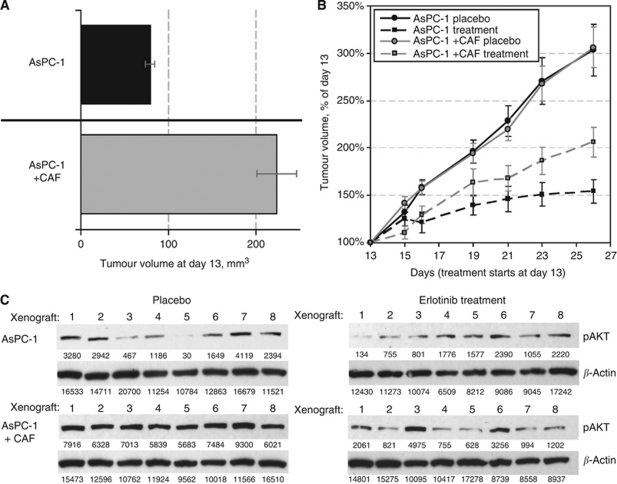
Effects of CAF on tumourigenesis and influence of erlotinib treatment on AKT activation. (**A**) Tumour volume difference between AsPC-1+CAF and AsPC-1 alone xenogratfs on day 13 post-inoculation. (**B**) Comparison of relative tumour growth between four groups of mice. All data points are represented by mean values with bars showing s.e.m. (**C**) Western blot analysis of AKT activation in eight representative tumours from each of the four groups of mice. Numbers underneath each lane represent the absolute band intensity.

Using the CAF:AsPC-1 xenograft model described above, we analysed the influence of CAF exert on xenograft response to anti-EGFR therapy with erlotinib, replicating our *in vitro* experiment design. As described, on day 13 after tumour inoculation, 2 : 1 CAF:AsPC-1 xenografts reached a larger size than their counterparts, which contained AsPC-1 cells alone (221 and 81 mm^3^, respectively, *P*=2 × 10^−7^) (Figure 7A). Erlotinib significantly inhibited tumour growth in both CAF:AsPC-1 and AsPC-1 xenografts, but the degree of inhibition was significantly less pronounced in the CAF-containing xenografts (*P*=0.008) (Figure 7B). CAF:AsPC-1 xenografts remained twice as large as AsPC-1 tumours, indicating an association between the presence of CAF and resistance to erlotinib therapy *in vivo*. After completion of treatment, animals were killed and xenografts were harvested. Protein analysis of AKT activation was performed in eight tumours from each animal treatment group (Figure 7C). We performed quantification of the immunoblots and normalised pAKT intensities to *β*-actin levels for each tumour. As expected, AKT phosphorylation levels in AsPC-1 xenografts were much lower than in CAF:AsPC-1 tumours (2298 average intensity *vs* 9641, respectively). These findings confirm that larger tumour sizes in the CAF-containing group are due, at least in part, to increased AKT signalling, further supporting active cross-talk mechanisms between CAF and carcinoma cells. Erlotinib was only able to partially abrogate the activation of AKT with higher levels of residual pAKT in CAF-containing tumours (2929 average intensity *vs* 2336, respectively).

**Figure 8:**
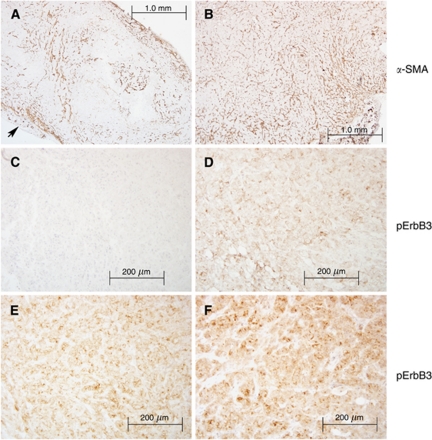
IHC analysis of xenograft tumours with and without CAF and effect of erlotinib treatment. The photographs (**A**, **B**) show low power (4 × ) representative sections of *α*-SMA staining of AsPC-1 and AsPC-1+CAF xenografts, respectively. The photographs (**C**, **D**) show representative sections of pErbB3 expression in erlotinib-treated AsPC-1 and AsPC-1+CAF xenografts. A high power (20 × ) view demonstrates positive membranous pattern, scored as intensity 2 on a 0–3 scale (**D**), the opposite (**C**) shows negative staining. The photographs (**E**, **F**) show representative sections of pErbB3 staining in placebo-treated AsPC-1 and AsPC-1+CAF xenografts.

We also estimated the overall fibroblast content of tumours using *α*-SMA immunohistochemistry. While *α*-SMA does stain additional elements such as murine blood vessels (Figure 8A, arrow), positive spindle cells were readily detected and identified as fibroblasts. Among tumours with admixture of CAF and carcinoma cells, the distribution of *α*-SMA-positive fibroblasts showed a uniform pattern (Figure 8B) as opposed to a ‘patchy’ pattern for the carcinoma cell only group (Figure 8A), where parts of tumour were almost devoid of fibroblasts. There was no overall difference in the total *α*-SMA-positive cell content between the two types of xenograft. In both cases, epithelial cells appeared morphologically identical without evidence of tumour necrosis. Phosphorylated ErbB3 protein localised to the membrane of carcinoma cells levels with lower expression levels in their cytoplasmic compartment (data not shown). Overall expression of pErbB3 protein remained higher in CAF-containing xenografts, and this pattern persisted following erlotinib treatment (average histoscore of 25 *vs* 10, CAF:AsPC-1 and AsPC-1 alone xenografts, respectively; Figure 8C–E). Tumour cell-surface localisation was present in contiguous areas of the tumour, often in the mid-sections between tumour surface and centre. These *in vivo* findings support the concept that CAF are diffusely present in our xenograft model providing a closer resemblance to the pronounced desmoplastic reaction seen in human PDAC specimens. Cancer-associated fibroblasts thus maintain a close interaction with carcinoma cells promoting PI3K/AKT signalling while contributing to an ‘escape’ mechanism from the effects of erlotinib in PDAC.

### MM-121 inhibits proliferation *in vitro* and *in vivo* and disrupts PI3K/AKT signalling

**Figure 9:**
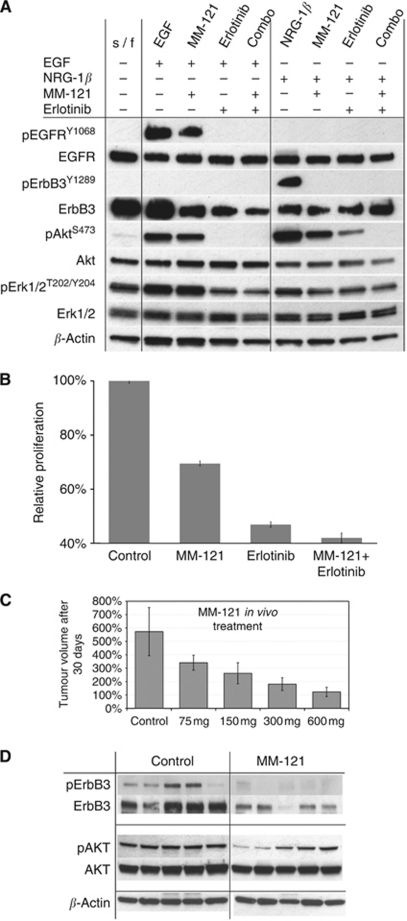
Effects of MM-121 ErbB3 antibody on AsPC-1 cell proliferation and signalling both *in vitro* and *in vivo*. (**A**) Western blot analysis of inhibitory effect of erlotinib, MM-121 and their combination following EGF or NRG-1 stimulation. (**B**) Inhibition of AsPC-1 proliferation with erlotinib, MM-121 and their combination. (**C**) MM-121 inhibits AsPC-1 tumour progression in a dose-dependent manner. (**D**) Western blot analysis of AsPC-1 xenografts depicting the inhibition of ErbB3 activation and expression and inhibition of pAKT.

We used a clinical grade ErbB3 Ab inhibitor (MM-121) to effectively halt AsPC-1 proliferation and ErbB3 downstream signalling *in vitro* (Figure 9). Pretreatment with MM-121 followed by NRG-1 stimulation rendered ligand-induced ErbB3 activation ineffective and combination of MM-121 with erlotinib completely abolished AKT activation in pancreatic cancer cells (Figure 9A). MM-121 also significantly decreased proliferation of AsPC-1 cells *in vitro* displaying an additive effect with the anti-proliferative properties of erlotinib (Figure 9B). *In vivo*, MM-121 effectively inhibited tumour growth of AsPC-1 subcutaneous xenografts in a dose-dependent manner. Following tumour harvest, protein analysis of MM-121-treated xenografts revealed marked inhibition of ErbB3 protein activation and expression with the consequent inhibition of AKT phosphorylation (Figure 9C and D).

## Discussion

PDAC remains an extremely aggressive cancer with a dismal clinical outcome. As traditional chemotherapeutic agents have proven to be largely ineffective, the therapeutic focus has shifted to molecular targeted therapy. Epidermal growth factor receptor signalling seems to have an important role in pancreatic cancer tumour progression and EGFR, at least in theory, has been deemed an attractive molecular target for pancreatic cancer treatment (Fjallskog *et al*, 2003). Growing knowledge suggests, however, that EGFR-mediated tumourigenic mechanisms as well as EGFR-targeted therapeutic approaches are very different for this tumour type when compared with better understood NSCLC or colorectal cancers. The utilisation of both small molecule and mAb anti-EGFR agents in combined therapeutic strategies for PDAC has been associated with little or no success (Xiong *et al*, 2004; Moore *et al*, 2007; Arnoletti *et al*, 2011). It has thus become evident that single EGFR inhibition is typically not sufficient to abort critical signalling pathways, as PDAC is not addicted solely to this pathway due to lack of gain-of-function *EGFR* mutations or *EGFR* genomic gain (Tzeng *et al*, 2007a, 2007c).

An alternative explanation for the modest results of EGFR molecular targeting in this type of tumour is the fact that PDAC is a complex, heterogeneous neoplasm that relies on multiple signalling pathways to promote tumour cell proliferation and invasion. One of the suggested mechanisms of resistance involves EGFR-independent activation of downstream signalling. The high incidence of oncogenic *KRAS* activating mutations found in PDAC (90% of tumours) has been proposed as a contributing factor to the limited effects of EGFR targeting (Almoguera *et al*, 1988). There is evidence that *KRAS* mutations are highly specific negative prognostic factors of response (*de novo* resistance) to EGFR-targeted therapy in both NSCLC and colorectal cancer (Bokemeyer *et al*, 2009; Jackman *et al*, 2009). However, a recent meta-analysis has shown low sensitivity and relatively high negative likelihood ratio of *KRAS* mutations for determining non-responsiveness, clearly showing that additional mechanisms of resistance to EGFR inhibitors exist (Linardou *et al*, 2008). The true role of *KRAS* mutations in regards to EGFR therapy for PDAC is yet to be fully discovered and careful cancer-type distinction must be employed as there is little evidence demonstrating true prognostic power of this clinical marker. Data available from the Canadian phase III trial suggest that the predictive nature of *KRAS* mutations to anti-EGFR treatments might not transfer to PDAC (da Cunha Santos *et al*, 2010). At the present time, it seems premature to dismiss treatment modalities, which include EGFR inhibition based solely on the high incidence of *KRAS* mutations found in PDAC. A novel approach is needed where a thorough understanding of the underlying mechanisms of resistance is employed to identify new targets for rational combined therapy strategies that may optimise patient selection and clinical results. In that context, we hypothesised that the PDAC stromal component may influence ErbB-mediated signalling with resultant critical effects on tumour cell proliferation and response to EGFR-targeted agents. We understand that this proposed mechanism could be just one of the many plausible scenarios explaining resistance to EGFR-targeted therapies since alternative escape routes such as TGF-*β* and IL-6 axis have been implicated in other tumour types (Yao *et al*, 2010). We believe, however, that PDAC has distinct biologic features compared with other malignancies, and this uniqueness applies to EGFR/ErbB3 interactions. Here, we present a first attempt to demonstrate the role of combined EGFR/ErbB3 inhibition in the attempt to disrupt stromal–epithelial interactions that promote PDAC cellular proliferation.

A recent consensus report on pancreatic cancer treatment acknowledges and emphasises the need to identify and validate relevant targets in the PDAC microenvironment, including stromal cells (Philip *et al*, 2009). Given the extent of PDAC desmoplasia, CAF constitute a major component of the tumour-associated stroma and several previous studies have identified these cells as active interactors of carcinoma cells. Cancer-associated fibroblasts have been shown to increase *in vitro* tumour cell proliferation, migration, invasion and colony formation, as well as *in vivo* tumour size and metastasis (Hwang *et al*, 2008). In our present study, we have incorporated stromal CAF to our pancreatic cancer xenografts to better model the real-life microenvironment found in human PDAC.

In search of the mechanisms that promote tumourigenic ErbB signalling, we have previously demonstrated that unlike NSCLC, pancreatic cancer cells lack activating *EGFR* mutations (Tzeng *et al*, 2007a). *EGFR* gene amplification, while prevalent in PDAC specimens, has been inconsistently linked with clinical response to EGFR inhibition in other tumour types (Tzeng *et al*, 2007b; Dahabreh *et al*, 2010). We, therefore, turned our attention to receptor heterodimerisation and ligand overexpression as plausible mechanisms of aberrant ErbB activation and downstream signalling.

ErbB3 overexpression has been clearly implicated in epithelial cancer progression (Kraus *et al*, 1989). It was originally felt that since the ErbB3 catalytic domain shows very weak kinase activity if any, ErbB3 was an obligate heterodimerisation partner (Berger *et al*, 2004). It has been recently demonstrated, however, that ErbB3 not only has kinase activity but its intracellular domain is competent to bind ATP and capable of catalysing auto-phosphorylation, a concept that is still being debated (Shi *et al*, 2010). NRG-1 as an ErbB3 ligand interacts with the receptor and increases its phosphorylation (Holbro *et al*, 2003). It has also been shown that the EGF-like domain of NRG-1 binds to domains I and II of ErbB3 leading to the activation of the receptor (Singer *et al*, 2001) For downstream signalling, ErbB3 couples with PI3K to activate the AKT signalling cascade (Kim *et al*, 1994; Soltoff *et al*, 1994). ErbB3 signalling promotes resistance to tyrosine kinase inhibition in breast cancer cells via heterodimerisation with ErbB2 (Sergina *et al*, 2007; Schoeberl *et al*, 2009). There is therefore considerable evidence that points to ErbB3 as a key hub within the cellular ErbB signalling network (Schoeberl *et al*, 2009).

Our prior findings indicate that pancreatic cancer cells sensitive to EGFR-targeted therapy can be rendered resistant via siRNA-induced ErbB3 inhibition, which suggests a critical influence of ErbB3 in pancreatic cancer EGFR signalling (Frolov *et al*, 2007). This role has been confirmed by the promotion of tumourigenesis employing stable ErbB3 transfection in the wild-type ErbB3-deficient pancreatic cancer cells (Tzeng *et al*, 2007b). We have also convincingly demonstrated that ErbB3 is the preferential heterodimerisation partner of EGFR in pancreatic cancer cells (Frolov *et al*, 2007). More importantly, however, ErbB3 is overexpressed in pancreatic cancer (Lemoine *et al*, 1992; Friess *et al*, 1995; Kolb *et al*, 2007) and high expression of ErbB3 correlates with advanced stage and decreased overall survival (Friess *et al*, 1995). ErbB3 ligands such as NRG-1 are expressed by pancreatic cancer cells and have been linked to pancreatic cancer cell growth and patient survival (Kolb *et al*, 2007). The influence of ligand expression on response to EGFR-targeted agents has already been described in colorectal cancer (Khambata-Ford *et al*, 2007; Jacobs *et al*, 2009). By analogy, and given the presence of EGFR-ErbB3 heterodimers in pancreatic cancer cells, we postulated that NRG-1, a specific ErbB3 ligand, is secreted by the stroma, forms an active paracrine loop and influences response to EGFR inhibition by erlotinib.

Our current results confirm that pancreatic cancer cell expression of ErbB3 and ErbB3-mediated signalling modulates response to EGFR inhibition. More importantly, aberrant pancreatic cancer ErbB3 signalling is a ligand-driven mechanism supported by CAF from the surrounding stroma as a source of NRG-1. Selective analysis of CAF from PDAC surgical specimens has allowed us to characterise a specific paracrine signalling cascade between stromal cells and carcinoma cells that promotes cancer cell proliferation *in vitro* and *in vivo*. We have identified the consistent expression of NRG-1 by CAF in the tumour microenvironment and have thoroughly demonstrated that this ligand promotes tumourigenesis through ErbB3/PI3K/AKT activation. Furthermore, we have demonstrated that CAF secretion of NRG-1 actively circumvents the inhibitory effect of erlotinib and promotes tumour growth providing an ‘escape’ mechanism to EGFR-targeted therapy. These findings may help explain the modest clinical results of EGFR-targeted therapy and support a role for combined EGFR/ErbB3 targeting in the treatment of advanced pancreatic cancer. They also provide further evidence that pancreatic CAF exhibit unique characteristics that distinguish them from other types of fibroblasts and support a very active stromal role in this dynamic tumour system. NRG-1 ligands are consistently expressed within the PDAC stroma and we propose ligand-driven ErbB signalling as a fundamental mechanism for tumour progression in PDAC. Finally, we have demonstrated for the first time the effectiveness of MM-121, a specific anti-ErbB3 inhibitor, to inhibit proliferation of PDAC cells as well as murine pancreatic cancer xenografts and to disrupt the activation of PI3K/AKT as a critical proliferative pathway.

Our results point to the utility of incorporating stromal elements to PDAC *in vivo* experimental models in an effort to better model real-life tumour microenvironment conditions. The inclusion of primary CAF in PDAC xenografts allows for dynamic stromal–epithelial interactions that beyond doubt exert a powerful influence on therapeutic response to targeted agents.

Despite our focus on ErbB3 ligands, it is highly likely that additional ligands are secreted by the stroma and that both ErbB and non-ErbB pathways are stimulated by that interaction. As mentioned, PDAC is a complex tumour with an array of synchronously activated pathways. Inhibition of ErbB signalling, even when targeting both EGFR and ErbB3, may not be sufficient to effectively halt PDAC tumour progression. Alternative non-ErbB-mediated escape mechanisms may be present or subsequently develop that render this type of approach ineffective. Additional targeted agents or optimal combinations with traditional chemotherapeutic drugs may be, therefore, necessary to achieve better treatment results in this challenging tumour type.

We conclude that stromal CAF-secreted NRG-1 stimulates ErbB3/AKT signalling and promotes pancreatic cancer cell tumourigenesis, providing a mechanism that may contribute to PDAC resistance to anti-EGFR therapy. We propose NRG-1 and ErbB3 as novel molecular targets that may lead to more successful therapeutic modalities for PDAC patients. We also suggest that MM-121 shows great potential in inhibiting PDAC ErbB3 signalling and should be tested in the clinical setting.
